# Evidence for a thromboembolic pathogenesis of lung cavitations in severely ill COVID-19 patients

**DOI:** 10.1038/s41598-021-95694-0

**Published:** 2021-08-06

**Authors:** Jan Matthias Kruse, Daniel Zickler, Willie M. Lüdemann, Sophie K. Piper, Inka Gotthardt, Jana Ihlow, Selina Greuel, David Horst, Andreas Kahl, Kai-Uwe Eckardt, Sefer Elezkurtaj

**Affiliations:** 1 Department of Nephrology and Medical Intensive Care, Charité – Universitätsmedizin Berlin, Augustenburger Platz 1, 13353 Berlin, Germany; 2 Institute of Radiology, Charité – Universitätsmedizin Berlin, Berlin, Germany; 3 Institute of Pathology, Charité – Universitätsmedizin Berlin, corporate member of Freie Universität Berlin and Humboldt-Universität zu Berlin, Charitéplatz 1, 10117 Berlin, Germany; 4 Institute of Biometry and Clinical Epidemiology, Charité – Universitätsmedizin Berlin, corporate member of Freie Universität Berlin and Humboldt-Universität zu Berlin, Charitéplatz 1, 10117 Berlin, Germany

**Keywords:** Viral infection, Pathogenesis

## Abstract

Severe acute respiratory syndrome coronavirus 2 (SARS-CoV-2) causing coronavirus disease 2019 (COVID-19) induces lung injury of varying severity, potentially causing severe acute respiratory distress syndrome (ARDS). Pulmonary injury patterns in COVID-19 patients differ from those in patients with other causes of ARDS. We aimed to explore the frequency and pathogenesis of cavitary lung lesions in critically ill patients with COVID-19. Retrospective study in 39 critically ill adult patients hospitalized with severe acute respiratory syndrome coronavirus 2 including lung injury of varying severity in a tertiary care referral center during March and May 2020, Berlin/Germany. We observed lung cavitations in an unusually large proportion of 22/39 (56%) COVID-19 patients treated on intensive care units (ICU), including 3/5 patients without mechanical ventilation. Median interquartile range (IQR) time between onset of symptoms and ICU admission was 11.5 (6.25–17.75) days. In 15 patients, lung cavitations were already present on the first CT scan, performed after ICU admission; in seven patients they developed during a subsequent median (IQR) observation period of 48 (35–58) days. In seven patients we found at least one cavitation with a diameter > 2 cm (maximum 10 cm). Patients who developed cavitations were older and had a higher body mass index. Autopsy findings in three patients revealed that the cavitations reflected lung infarcts undergoing liquefaction, secondary to thrombotic pulmonary artery branch occlusions. Lung cavitations appear to be a frequent complication of severely ill COVID-19 patients, probably related to the prothrombotic state associated with COVID-19.

## Introduction

The novel severe acute respiratory syndrome coronavirus 2 (SARS-CoV-2), a beta coronavirus is causing coronavirus disease 2019 (COVID-19). In the past months, its spread to most countries of the world has led to a global pandemic^1^, causing more than 1,600,000 deaths to date^2^.

Severe hypoxemic respiratory failure due to acute lung injury is the most common complication leading to admission to intensive care units (ICU) and one of the main causes of death^3,4^. The precise etiology of the impaired gas exchange and optimal treatment strategies remain a matter of debate^5^. Of note, some clinical aspects seem to differentiate COVID-19 from other forms of acute respiratory distress syndrome (ARDS). In particular, in many COVID-19 patients with ARDS the pulmonary compliance is not significantly altered, in contrast to classic ARDS^5^.

Besides lung injury a prothrombotic state has emerged as an important characteristic of COVID-19. Data from both clinical studies and postmortem case series demonstrate a high incidence of thromboembolic events^6–9^. These events include pulmonary artery occlusions, which may have a thrombotic or thromboembolic origin^10^. Massive pulmonary embolism has been suggested to cause out-of-hospital mortality^11^. Whether pulmonary artery occlusion contributes to hypoxemic respiratory failure due to impaired lung perfusion and dead space ventilation is controversial^12^.

To clarify the etiology of the seemingly high incidence of cavitary lesions was the purpose of the retrospective analysis of our cohort of severely ill COVID-19 patients.

## Material and methods

Since we conducted a retrospective analysis the materials and methods section describes the standard of care of COVID-19 patients in our institution. We screened the electronic records for possible discriminating factors between patients who did and did not delvelope cavitary lesions with special consideration of markers of the coagulation und inflammatory cascades.

### Patients

We retrospectively analyzed clinical patient data of 39 COVID-19 patients admitted to ICU between March and May 2020 who received at least one chest CT shortly before or during their ICU stay. All patients were tested positive for SARS-CoV-2 by polymerase chain reaction (PCR). Five patients were admitted through the emergency department, one patient was directly admitted to our ICU from an outpatient setting, two patients had worsened during their stay on regular wards and 31 patients were secondary referrals from other ICUs.

### Anticoagulation

All patients received unfractionated heparin (UFH) with a targeted partial thromboplastin time (PTT) of 50–55. Patients who suffered from venous thromboembolic (VTE) complications or who had other indications for therapeutic anticoagulation were dosed for a target PTT of 60–80 s.

Patients who did not reach the target PTT with usual doses of UFH were switched to Argatroban. Patients were also switched to Argatroban when they required extracorporeal membrane oxygenation therapy (ECMO).

### Mechanical ventilation

All mechanically ventilated patients received pressure controlled ventilation. Positive endexspiratory pressure (PEEP) was titrated to reach best possible oxygenation index. Patients received low tidal volume ventilation with a target tidal volume (VT) of 6 ml/kg/PBW and a diving pressure below 15 mmHg was targeted.

### Data collection

CT scans were analyzed independently by two of us (JMK, WML) for parenchymal cavities, defined as a lucency within a zone of pulmonary consolidation, a mass, or a nodule; hence, a lucent area within the lung that may or may not contain a fluid level and that was surrounded by a wall of varied thickness^13^.

Screening for venous thrombosis was performed using ultrasound (GE Vivid S70 ultrasound machine with a 9L-D probe) in all patients after ICU admission and repeated at least weekly.

Laboratory parameters included viscoelastic coagulation testing after ICU admission using the ROTEM Sigma System (Tem International, Munich, Germany)^14^. Maximum values of C-reactive protein (CRP), d-dimers, fibrinogen, leukocytes, interleukin-6 and procalcitonin were compared between patient groups.

Microbiology reports of all of the collected specimens from the respiratory tract and blood cultures were analyzed for pneumopathogenic species.

The highest levels of PEEP, peak inspiratory pressure and driving pressure during the ICU stay were recorded and analyzed in ventilated patients.

The highest values for SOFA and APACHE II during the course of therapy were recorded and analyzed.

Five patients (13%) had died during their ICU stay, 22 patients (56%) had been discharged alive and 12 patients (31%) were still treated on ICU. On three of the five patients who succumbed, autopsies were performed on days 31, 37 and 47 after ICU admission. The study was approved by the Ethics Committee of the Charité (EA4/115/20) and was in compliance with the Declaration of Helsinki.

### Autopsy procedure

Complete autopsies and tissue sampling were performed by opening all luminal structures and lamellar incisions of all parenchymatous organs. The lung was dissected by a combination of lamellar incision and subsequent selective preparation of the airways and blood vessels to allow for visualization of pulmonary lesions in the context of vascular supply and airways. For histopathology, representative tissue samples of all organs were fixed in 4% buffered formalin, dehydrated, paraffin embedded and sectioned with a thickness of 4 µm. Paraffin sections were stained with hematoxylin and eosin (HE), periodic acid Schiff´s reaction (PAS), Van Gieson’s elastic stain, Prussian blue stain and Kongo-red stain. Two pathologists (DH and SE) examined all slides by light microscopy.

### Statistics

Statistical evaluations were performed with IBM SPSS Statistics Version 26 (New York, USA). The descriptives are given as median and limits of the interquartile range [IQR] for continuous variables or as absolute and relative frequencies for categorical variables.

Due to the retrospective nature of the study no sample-size or power calculations were performed. We conducted a post-hoc power calculation as a substitute.

Mann–Whitney *U* tests were used to compare differences between patient groups in continuous variables while Chi-squared tests were used for categorical data. A two-sided significance level of 0.05 was applied without adjustment for multiple comparison. All *p*-values constitute exploratory data analysis and do not allow for confirmatory generalization of results.

### Ethics approval and consent to participate

The study was approved by the ethics committees of Charité – Universitätsmedizin Berlin (EA4/115/20) and was in compliance with the Declaration of Helsinki. Informed consent was waived by the ethics committees of Charité – Universitätsmedizin Berlin due to the retrospective nature of the study.

## Results

### Chest CT findings

64 CT scans were analyzed, of which 39 were performed shortly before or after ICU admission and 25 during the course of the ICU stay. In 37/39 (95%) of patients we found ground glass opacities of the lung parenchyma, characterized as “mosaic pattern”. Cavitary lung lesions were found in 22 patients (56; 95% confidence interval 41–72%), of which 15 patients presented with cavitations in the initial CT while seven patients exhibited cavitary lesions in a subsequent CT scan.

**Figure 1:**
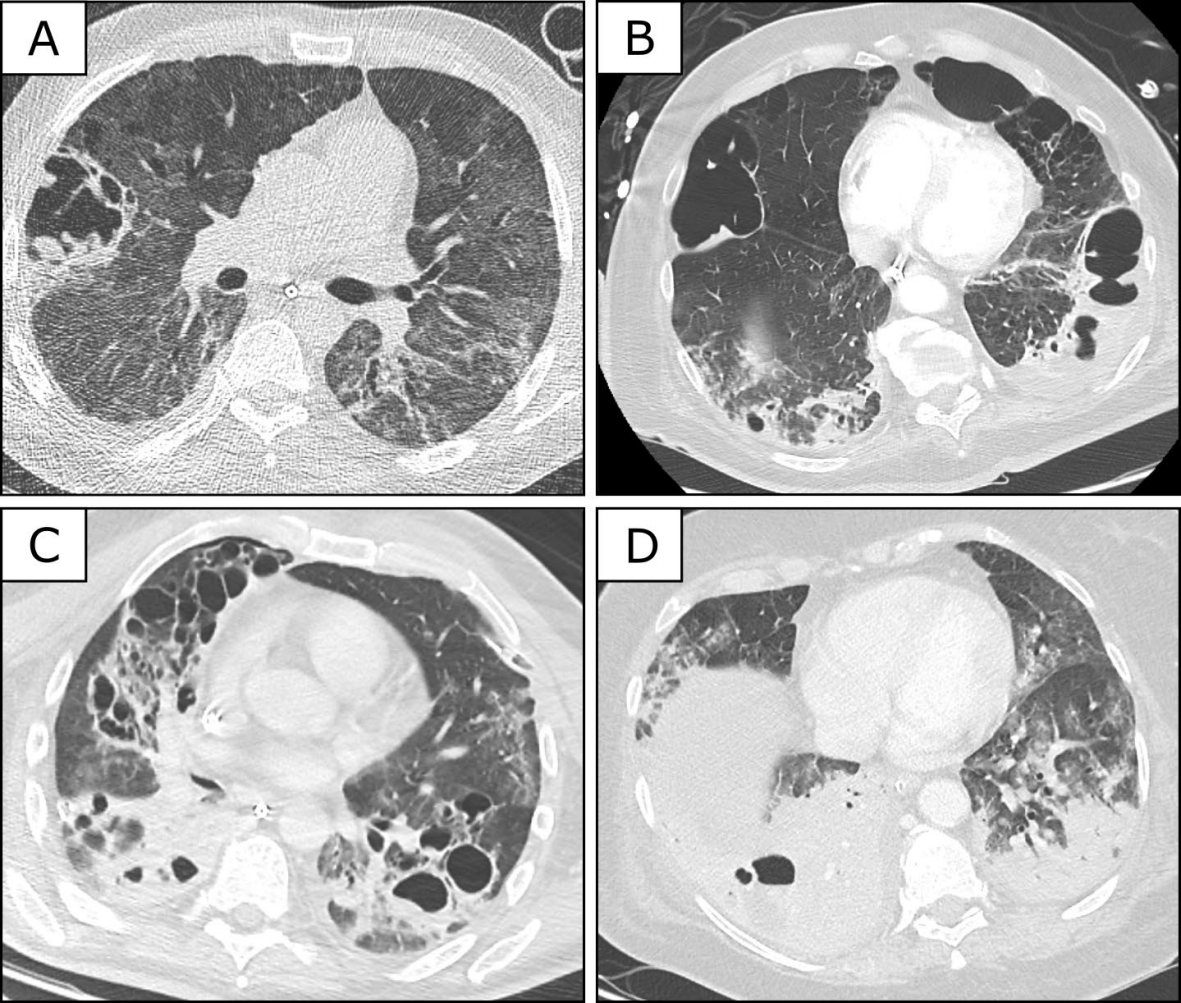
CT scans in COVID-19 patients receiving long term ICU care. CT scans showing typical cavitary lesions in four COVID-19 patients. (**A**) Single cavity in a 49-year old male patient 29 days after symptom onset; (**B**) bilateral cavitations in a 77-year old male patient 39 days after onset of symptoms; (**C**) extensive bilateral cavities in a 57-year old male patient on day 36 after symptom onset; (**D**) right-sided cavitation in a large lower lobe consolidation in a 69-year old female patient 29 days after onset of symptoms. Only open source software (GIMP 2.10.18 https://www.gimp.org/ and Inkscape, Version 1.0 (4035a4fb49, 2020–05-01) ) were used to generate the figure.

Among patients with cavitations the number of cavities between left and right lung were similar. Cavitations were evenly distributed between central and peripheral parts of the lungs. Eleven patients presented with peripherally and 11 patients with centrally located cavitations. Thirteen patients showed involvement of the lower parts, while in 9 patients cavitations were found in the upper parts of the lung. Figure 1 shows typical characteristics of cavitary lesions on representative CT scans. The spectrum ranges from unilateral peripheral cavitations (Fig. 1A) to well-separated lesions in both lungs (Fig. 1B) and extensive bilateral cavitating destructions of the lung parenchyma (Fig. 1C). Maximum cavitation size in the two patients with the largest cavities was approximately 10 cm. Repetitive CT scans had been performed in 13/22 patients. In these patients a comparison between first and last CT scan showed an increase in cavity number in eight patients, no change in four patients and a decline in one patient. In 18/22 patients, cavities were identified in opaque lung areas (example in Fig. 1D). Among patients with lung cavities 19 were on mechanical ventilation, while three patients required no mechanical ventilation.

### Clinical characteristics

Table 1 shows demographics, preexisting comorbidities and treatment parameters of the patient cohort and the two subgroups with and without lung cavitations. Most parameters were similar between groups, except that patients in the group with cavitations were older and had a higher body mass index (BMI) than those without cavitations.

**Table 1:** Patient characteristics of total cohort and the groups with and without cavitary lesions on CT scan.

	Cohort (N = 39)		Lung cavitations (N = 22)		No cavitations (N = 17)		*p*-value
Age (years, (median, [IQR]))	67	[58–76]	69.5	[60.5–77.3]	62	[61.5–67.75]	0.047
Gender, male (n, %)	34	(87%)	19	(86%)	15	(88%)	0.86 s
BMI, kg/m^2^ (median, [IQR])	28	[25–33]	27.8	[24.2–33]	25.2	[24.32–28.7]	0.009
Days between symptom onset and ICU admission	11	[25–33]	12	[6–17]	11	[6–19]	0.49
Duration ICU stay, days (median, [IQR])	28	[15.5–39.8]	30.9	[27.07–34.25]	25	[8.5–30.5]	0.25
Intubation (n, %)	34	(87.2%)	18	(81%)	16	(94%)	0.47
ECMO (n, %)	10	(25.6%)	5	(22%)	5	(29.4%)	0.22
CRRT* (n, %)	19	(48.7%)	12	(54.5%)	7	(50%)	0.19
SOFA-Score (median, [IQR])	9	[7–12]	10	[6–11]	9	[7–11]	0.49
APACHE-Score (median, [IQR])	28	[22–33]	28	[24–34]	26	[22–34]	0.65
Secondary ICU referral	23	(59.0%)	11	(48%)	12	(52%)	0.32
PEEP (median, [IQR])	17	[15–18]	17	[15–18]	17	[15–19]	0.58
PIP (median, [IQR])	31	[29–35]	32	[27–35]	31	[29–34]	0.90
Delta P (median, [IQR])	15	[12–17]	17	[15–18]	15	[12–16]	0.86
**Preexisting conditions**							
Coronary artery disease (n, %)	9	(23.1%)	7	(31.2%)	2	(11.7%)	0.44
Hypertension (n, %)	27	(74.0%)	14	(63.63%)	13	(76.5%)	0.12
Diabetes mellitus/insulin resistance (n, %)	14	(35.9%)	8	(36.4%)	6	(35.3%)	0.62
Chronic kidney disease (n, %)	7	(17.9%)	3	(13.6%)	4	(23.5%)	0.42
Chronic dialysis (n, %)	1	(2.5%)	0	(0%)	1	(5.9%)	0.30
COPD (n, %)	10	(25.6%)	7	(31.8%)	3	(17.6%)	0.32

ECMO, Extracorporeal Membrane Oxygenation; SOFA, Sequential Organ Failure Assessment; CRRT, Continuous Renal Replacement Therapy; APACHE, Acute Physiology And Chronic Health Evaluation. PEEP, positive endexspirarory pressure, PIP, Peak inspiratory pressure, deltaP, driving pressure.

*Until the time point, when the study was censored.

Fifteen patients (38%) developed deep vein thrombosis. Pulmonary emboli were identified by CT imaging in five patients (12% of the whole group), four with and one without lung cavitations. Two patients had an ischemic stroke (5%). One patient required urgent extracorporeal membrane oxygenation (ECMO)-circuit change due to fulminant clotting and one patient showed acute thrombotic obstruction of the venous drainage cannula on ECMO. There was no statistically significant difference when considering all these thromboembolic complications combined between the group of patients with and without lung cavities.

### Ventilation parameters

34 patients (87%) were mechanically ventilated via endotracheal tube or tracheostomy using a pressure controlled mode. The median peak inspiratory pressure (PIP) was 31 mbar [IQR 28.7–35.0], the median positive endexspiratory pressure was 17 mbar [IQR 15–18.2]. The median driving pressure was 15 mbar [IQR 11.7–17.2]. There were no significant differences in these parameters between the group of patients with and without cavitary lesions on CT. 25 patients were proned (64%), 12 patients in the group with and 13 patients in the group without lung cavitations. Ten Patients received nitric oxide (26%), six and four patients with and without cavitations, respectively. Ten patients were treated with veno-venous ECMO (26%), four and six patients with and without cavitations, respectively.

### Laboratory and microbiology findings

Table 2 shows markers of inflammation and coagulation. These parameters did not differ significantly between the patients with and without cavitations.

**Table 2:** Coagulation, inflammatory and ROTEM parameters of total cohort and sub cohorts with and without cavitary lesions on CT-scan.

	Cohort (N = 39)						
	Median	[IQR]	Cavitations				
			Yes (N = 22)		No (N = 17)		*p*-value
			Median	[IQR]	Median	[IQR]	
**Laboratory variables (normal values)**							
Haemoglobin (12.5–17.2 g/dL)	10.1	[8.5–11.2]	10.1	[8.7–11.9]	9.50	[8.1–10.9]	0.34
White blood cells (3.5–10.5/nl)	19.3	[16.3–28.8]	21.4	[17–30.6]	19.3	[14.9–28.8]	0.63
Platelet count (150–370/nl)	186	[131.3–314]	190	[128–254.0]	186	[121–324]	0.68
Prothrombin time (70–130%)	71	[62–82.7]	70	[51–82.5]	74.5	[67.25–85]	0.27
INR (0.9–1.25)	1.35	[1.2–1.6]	1.5	[1.3–1.7]	1.29	[1.1–1.4]	0.12
PTT (26–40 s)	45.7	[40–56.1]	45.1	[40.3–585]	46.1	[39.1–56]	0.75
Fibrinogen (1.6–4 g/l)	6.6	[4.7–7.8]	6.4	[4.7–7.2]	6.1	[4.6–7.9]	0.38
D-dimers (< 0.5 mg/l)	8.4	[3.9–17.2]	10	[3.6–18.6]	8.2	[3.9–11.6]	0.48
Procalcitonin (0.5 µg/l)	7.6	[1.9–15.7]	7.9	[1–16.9]	7.6	[3.1–18.9]	0.86
CRP (< 0.5 mg/l)	312.9	[207.1–344.1]	305.6	[181.8–352.5]	333.9	[215.6–344.4]	0.56
IL-6 (< 7 ng/l)	558.6	[180.2–1921.7]	567.2	[163.8–18600]	550	[179.5–1894.5]	0.97
Ferritin (30–400 µg/l)	2619.8	[1557–7111.9]	2621	[1155.7–8068.8]	2113.3	[1710.9–6392.2]	0.50
Ristocetin-Co-factor (%)	349	[204–404]	349	[179.5–429.5]	291	[259–401.5]	0.78
vWillebrand antigen (%)	391	[283.5–400]	394	[266.5–400]	368.5	[278.7–400]	0.96
Factor VIII (50–150%)	258	[190.5–319.5]	259	[212.2–293.5]	245	[153.2–340]	0.71
EXTEM MCF (mm)	75	[70–78]	74.5	[69.7–78.2]	76	[70.5–77.5]	0.89

Positive cultures of respiratory secretions were found in 32 of our patients (82%), 16 (73%) patients with and 16 patients (94%) without pulmonary cavities. The microbial spectrum appeared typical for ventilated patients. Bacterial species known to be able to induce pulmonary abscesses, such as *Klebsiella spp.* and *Staphylococus aureus* were cultivated in 13 and 11 of the patients with or without lung cavities, respectively. All broncheoalveolar lavage specimens were tested for Mycobacterium tuberculosis and none of them revealed a positive result.

### Autopsy findings

**Figure 2:**
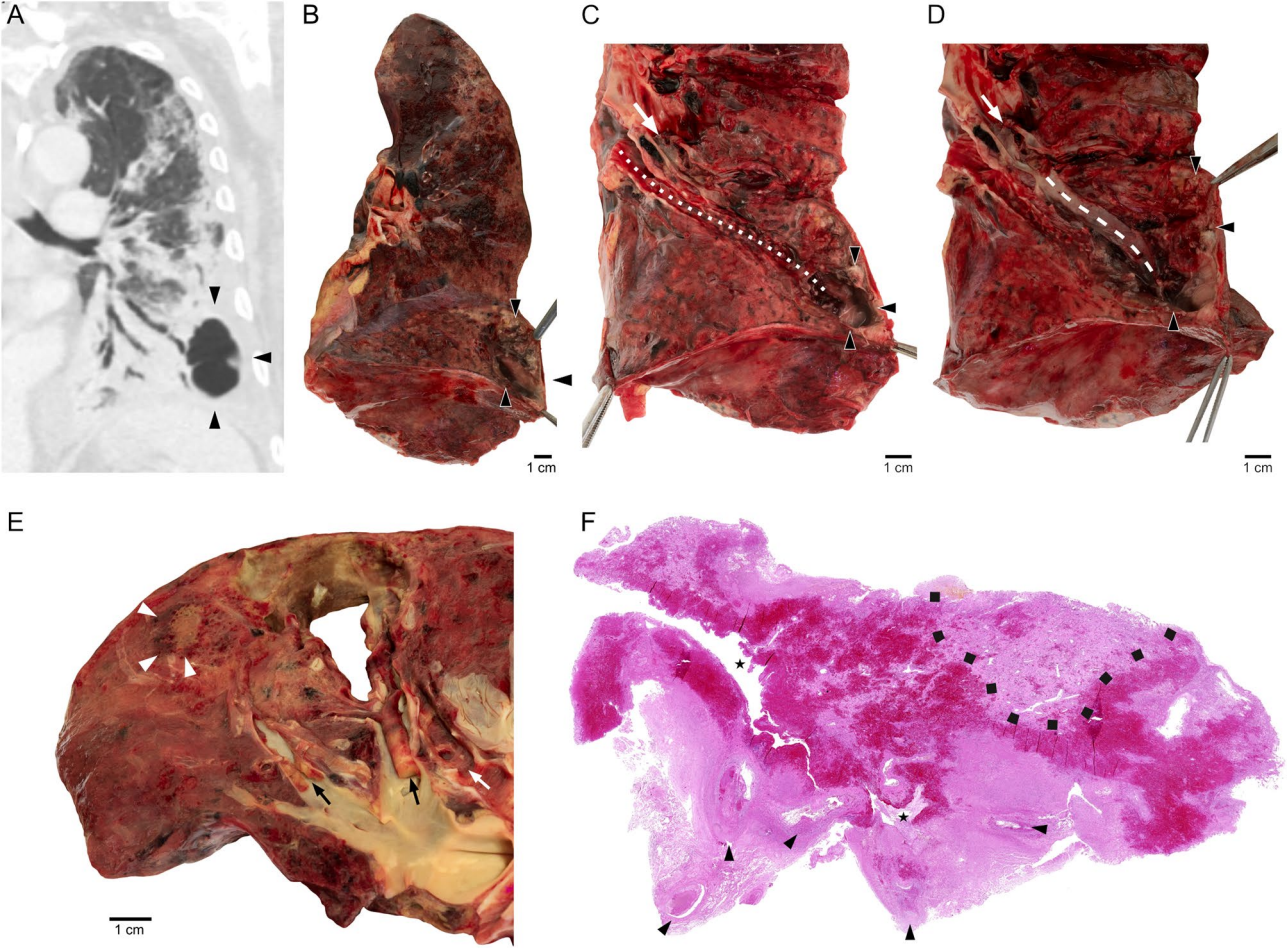
Postmortem findings in two patients. (**A**)–(**D**) show findings in a 77-year old patient who died 42 days after ICU admission. (**A**) Frontal reconstruction of lung CT showing large basal, left-sided cavity (corresponding to cross-sectional CT scan in Fig. 1B). Macroscopic findings during subsequent preparation steps show (**B**) opened lung cavity with necrotic lining (arrowheads), (**C**) direct connection of an opened bronchus with the cavity (dotted line), corresponding to positive aerogram on CT (**A**) and (**D**) a pulmonary artery branch (stippled line), directly connected with the necrotic cavity. A thrombotic vessel occlusion is indicated by white arrows. (**E**) and (**F**) show macro- and microscopic findings in a 69-year old patient, who died 33 days after ICU admission. (**E**) Opened pulmonary artery branches with subtotal (left) and total (right) occlusion of the vessel lumen (black arrows), and a directly adjacent lung cavity, suggesting that the large thrombus on the right has caused liquefactive infarct necrosis of the lung parenchyma. The necrotic area has gained access to the bronchial system (white arrow). The adjacent lung parenchyma shows a combined anemic and hemorrhagic infarct (arrowheads) that has not undergone cavitary transformation. (**F**) Histological sectioning (HE) shows multiple thrombotic occlusions (arrowheads) of pulmonary artery branches with consecutive anemic infarct necrosis (light red zones) with entrapment of bronchial airways (asterisks). Dark red zones around the bronchial airways represent hemorrhagic necrosis. The black dotted line delineates a small area of vital lung parenchyma.

In all three patients in whom an autopsy was performed, lung cavities had previously been identified on CT scans. External examination of the lungs showed pronounced pleural fibrin deposits and sunken lung areas, which corresponded to bullous transformations of lung parenchyma. Findings in two patients are presented in Fig. 2. Upon opening the cavities appeared as areas of liquified necrosis (Fig. 2B). Careful dissection revealed connections between cavities and bronchial system (Fig. 2C). Moreover, preparation of the pulmonary vessels on frontally oriented cross-sections yielded unequivocal associations of cavitary lesions with thrombotic occlusion of the supplying pulmonary artery branches (Fig. 2D, E). Microscopy of adjacent lung tissue revealed numerous thrombotic vascular occlusions and extended, partially hemorrhagic and partially anemic infarct zones in spatial association with vascular occlusions (Fig. 2F). The border zone of the infarct areas showed pronounced neutrophil infiltrations, but there was morphologic evidence for bacterial colonization. In summary, macro- and microscopic findings in combination suggested extensive vascular occlusions of different duration with multiple pulmonary infarctions of different size, some of which had transformed into liquefying necrosis, corresponding to large cavities. Table 3 shows markers of coagulation, inflammation and ventilation of the patients, who underwent autopsy.

**Table 3:** Coagulation, inflammatory and ventilatory parameters of three patients who underwent autopsy.

Patient	1	2	3
Prothrombin time (70–130%)	69	50	71
INR (0.9–1.25)	1.2	2	1.2
PTT (26–40 s)	46	64	38
Fibrinogen (1.6–4 g/l)	5.36	6.2	5.2
D-dimers (< 0.5 mg/l)	13.1	6.6	3.0
Procalcitonin (0.5 µg/l)	0.6	1.8	0.2
CRP (< 0.5 mg/l)	157	255	133
IL-6 (< 7 ng/l)	142	224	104
Ferritin (30–400 µg/l)	2633	4078	1490
EXTEM MCF (mm)	58	78	78
PEEP (mmHg)	17	18	16
PIP (mmHg)	35	41	31
deltaP (mmHg)	18	23	15

PEEP, positive endexspirarory pressure, PIP, Peak inspiratory pressure, deltaP, driving pressure, CRP, C-reactive-protein, IL-6, Interleukin-6, INR, International normalized ratio, PTT Partial Thromboplastin Time.

## Discussion

We noted extensive lung cavitations in single cases of COVID-19, which prompted us to perform a systematic analysis of a cohort of 39 critically ill COVID-19 patients consecutively admitted to two ICUs in a tertiary care referral center. This analysis revealed a high incidence of cavitary lung lesions. Computer tomography (CT) morphology and the results of postmortem macro- and micropathological examination point to an ischemic etiology of these lesions due to thrombotic obstruction of pulmonary artery branches.Our findings indicate that lung cavitations of variable size, at least in part a consequence of liquefying ischemic lung infarcts, contribute to lung pathology and loss of functional lung parenchyma in COVID-19 patients. These observations extend the reported spectrum of lung CT findings that is considered as typical for COVID-19, including ground glass opacities, consolidations and a “crazy paving pattern” of the lung parenchyma^15,16^.

In general, infectious causes and ventilator induced lung injury are recognized as the main etiologies of lung cavities in critically ill patients^17^. Ischemia is less commonly considered as a cause, although cavitations are described in up to 32% of patients with pulmonary embolism and are a common finding in patients suffering from chronic thromboembolic pulmonary hypertension^18,19^. While the precise pathogenesis is difficult to ascertain in individual cases in our study, we believe that several lines of evidence indicate that an ischemic pathogenesis rather than alternative causes play a major role.

First, several of our findings are not consistent with primarily ventilator induced lung injury. Ventilator settings were chosen to minimize lung trauma and did not differ significantly between the groups with and without lung cavities. The distribution of the cavities with a significant proportion of central lesions and involvement of the lower parts of the lung argues against ventilator induced lung injury, since from our experience one would expect mainly peripheral lesions in the upper lobes, if mechanical overdistension played the main role. The observation that a high percentage of lesions occurred in preexisting opacities also seems rather untypical for classical ventilator induced lung alterations. Due to higher compliance of the less affected regions, overdistension tends to occur preferentially in non-opaque regions of the lung. Most striking is the fact, that three of five patients, who did not need mechanical ventilation, also developed cavitations. Still mechanical ventilation might play an important role in the development of the cavitary lesions in that it aggravates the damage done by microvascular and macrovascular thrombosis. We therefore think that sticking to the principles of lung-protective ventilation is of great importance in this group of patients to minimize pulmonal destructions even if the lung compliance does not seem to be altered in every case.

Second, patients who developed cavitations did not exhibit positive cultures more often than the patients without, nor did we find typical abscess inducing species more frequently in the patients with lung cavities. However, a secondary bacterial infection of infarcted areas, transforming into cavitations cannot be ruled out.

Third, we found evidence for a prothrombotic state both in terms of coagulation parameters and thromboembolic complications. Although thromboembolic complications were evenly distributed between patients with and without lung cavities, notably pulmonary embolism was more frequent in patients with cavities (4 vs. 1). Patients who developed cavitations during the course of their disease were significantly older and had a higher BMI. Obesity has been associated with a prothrombotic state and hypofibrinolysis^20–24^. The “mosaic pattern” of the lung-parenchyma that we observed in accordance with previous publications and the lung cavities strikingly resemble findings in patients with chronic thromboembolic pulmonary hypertension^15,18,19^.

During the time of this observational study critical care resources for COVID-19 patients were in no way compromised in our regional setting, enabling long-term ICU therapy. Together with a very large proportion of secondary referrals this might explain why others have to the best of our knowledge not yet reported similar observations. However, several other observations support the concept of impaired lung perfusion in COVID-19 patients. Ackermann et al. found a high incidence of microvascular thrombi and signs of endothelitis during autopsy of seven COVID-19 patients^6^. Lang et al., using dual source computer tomography, discovered severe perfusion abnormalities in the lungs of three COVID-19 patients and postulated a significant contribution of altered perfusion to the etiology of respiratory failure in COVID-19^12^.

In terms of the mechanisms potentially causing pulmonary hypoperfusion, SARS-CoV-2 binds to the angiotensin converting enzyme *2* (ACE-2) receptor of alveolar epithelial cells^25^. There is evidence for consecutive downregulation of ACE-2 leading to increased levels of angiotensin II^25^. High levels of angiotensin II in the pulmonary circulation may lead to endothelial activation and vasoconstriction and promote a prothrombotic state^26^. Hypoperfusion of pulmonary artery branches either due to vasoconstriction or due to thromboembolic occlusion will lead to increased dead space ventilation and impaired gas exchange, consistent with the ventilation pattern observed in COVID-19^5,27^. Selective perfusion of the pulmonary circulation through anastomoses between the bronchial and the pulmonary circulation might further contribute to respiratory failure due to an increased right-to left shunt fraction^28^.

By implying a link between a prothrombotic stage, pulmonary hypoperfusion and structural lung changes, our data add to the considerations for therapeutic anticoagulation in COVID-19 patients. Interestingly in this regard Wang et al. recently reported a positive impact of tissue plasminogen activator treatment on oxygenation in a case series^29^. An association of higher therapeutic targets of systemic anticoagulation and improved survival has also been reported^11,30^. Although our data point towards a thromboembolic etiology of the cavitary lesions, the markers of coagulation measured in our study could not discriminate between the two groups and significant differences were only found regarding age and BMI. D-Dimers for example were not significantly different between the groups. One might argue that due to the high degree of activation of the coagulation system in both groups, the d-dimers are not sensitive enough to detect a difference. Increased age and body weight might lead to structural weakness of the lung tissue facilitating the development of cavitations. Hopefully future studies will identify more sensitive and specific laboratory markers in this regard and clarify the precipitating factors that add to the development of cavitary lung destruction in certain patient populations.

Our study has several limitations. Being retrospective and non-interventional it can only be hypothesis-generating. It is monocentric and focusses on severely ill patients, many of whom received ICU treatment for several weeks and our findings may not be generalizable to less severely ill patients. Due to the relatively small sample size, the power of our study is limited. We found a high proportion of cavitary lung lesions in the CT-scan (22/39 = 56%). The 95% confidence interval is 41–72% for our finding of this high incidence of lung cavitations. We included all 39 consecutive patients who received a CT-scan and were treated in our intensive care unit during the first COVID-19 wave, so we cannot provide a larger data set.

To make a more accurate assessment of the proportion of patients with cavitation we would have to include more patient data. For a sample size of 100, a two-sided 95% confidence interval for a single proportion using the large sample normal approximation would extend 0.097 from the observed proportion for an expected proportion of 0.56.

With a sample size of 380, a two-sided 95% confidence interval for a single proportion using the large sample normal approximation would extend only 0.05 from the observed proportion for an expected proportion of 0.56.

The etiology of lung cavitations is possibly heterogeneous and multifactorial. Finally, the number of autopsies supporting our interpretation is small and although all point to the exact same pathogenesis of the cavitations we do not know whether the same results would have been found in the lungs of the other patients of the cohort.

In conclusion, we found that cavitating lung lesions occur frequently in severely ill COVID-19 patients and provide evidence that pulmonary hypoperfusion and occlusion of pulmonary arteries plays an important role in the pathogenesis of these lesions.

## Conclusion

Although the small sample size limits the power of our study, we found a high incidence of cavitary lesions in our cohort of severely ill patients with COVID-19. Our findings underline the importance of sufficient anticoagulation in the management of patients with severe COVID-19 pneumonia. Furthermore our data show, how vulnerable the malperfused lungs of these patients are, even though the compliance might not be altered in the beginning. This points to the importance of lung protective ventilation in order to avoid further damage in the poorly perfused areas of the lungs of these patients.

## Data Availability

The datasets analyzed during the current study are available from the corresponding author upon reasonable request.

## Abbreviations

ACE-2: Angiotensin converting enzyme 2
APACHE: Acute Physiology And Chronic Health Evaluation
ARDS: Acute respiratory distress syndrome
BMI: Body mass index
COVID-19: Coronavirus disease 2019
CRP: C-reactive protein
CRRT: Continuous renal replacement therapy
CT: Computer tomography
ECMO: Extracorporeal membrane oxygenation therapy
HE: Hematoxylin and eosin staining
ICU: Intensive care unit
IQR: Interquartile range
PAS: Periodic acid Schiff’s reaction
PCR: Polymerase chain reaction
PEEP: Positive endexspiratory pressure
PIP: Peak inspiratory pressure
PTT: Partial thromboplastin time
SARS-CoV-2: Severe acute respiratory syndrome coronavirus 2
SOFA: Sequential Organ Failure Assessment
UFH: Unfractionated heparin
VT: Tidal volume
VTE: Venous thromboembolic
